# Evaluation of distance learning IMCI training program: the case of Tanzania

**DOI:** 10.1186/s12913-018-3336-y

**Published:** 2018-07-13

**Authors:** Lulu M. Muhe, Nemes Iriya, Felixambrose Bundala, Mary Azayo, Maryam Juma Bakari, Asia Hussein, Theopista John

**Affiliations:** 1 Department of Pediatrics and Child Health, Addis Ababa University, P.O.Box 1768, Addis Ababa, Ethiopia; 2 Child and Adolescent Health, World Health Organization Country Office, Dar Es Salaam, Tanzania; 3 Reproductive and Child Health Section- MOHCDGEC, Dar Es Salaam, Tanzania; 4 UNICEF Country Office, Dar Es Salaam, Tanzania; 5 MOH, Zanzibar, Tanzania

**Keywords:** Distance learning IMCI, Standard IMCI, Blending training, Peer learning

## Abstract

**Background:**

The standard 11-days IMCI (Integrated Management of Childhood Illness) training course (standard IMCI) has faced barriers such as high cost to scale up. Distance learning IMCI training program was developed as an alternative to the standard IMCI course. This article presents the evaluation results of the implementation of distance learning IMCI training program in Tanzania.

**Methods:**

From December 2012 to end of June 2015, a total of 4806 health care providers (HCP) were trained on distance learning IMCI from 1427 health facilities {HF) in 68 districts in Tanzania. Clinical assessments were done at the end of each course and on follow up visits of health facilities 4 to 6 weeks after training. The results of those assessments are used to compare performance of health care providers trained in distance learning IMCI with those trained in the standard IMCI course. Statistical analysis is done by comparing proportions of those with appropriate performances using four WHO priority performance indicators as well as cost of conducting the courses. In addition, the perspectives of health care providers, IMCI course facilitators, policy makers and partners were gathered using either focussed group discussions or structured questionnaires.

**Results:**

Distance learning IMCI allowed clusters of training courses to take place in parallel, allowing rapid expansion of IMCI coverage. Health care providers trained in distance learning IMCI performed equally well as those trained in the standard IMCI course in assessing Main Symptoms, treating sick children and counselling caretakers appropriately. They performed better in assessing Danger Signs. Distance learning IMCI gave a 70% reduction in cost of conducting the training courses.

**Conclusion:**

Distance learning IMCI is an alternative to scaling up IMCI as it provides an effective option with significant cost reduction in conducting training courses.

**Electronic supplementary material:**

The online version of this article (10.1186/s12913-018-3336-y) contains supplementary material, which is available to authorized users.

## Background

Training evaluation is the systematic collection of data regarding the success of training programs [1]. One of the learning outcomes of training is the acquisition of technical or motor skills. In the area of health, training evaluation is a systematic collection of data evaluating the performance of health care providers (HCP) in assessing and treating patients. In this article we describe the evaluation of a distance learning program on child health, specifically, the Integrated Management of Childhood Illness (IMCI), hereafter referred as distance learning IMCI.

IMCI is a holistic training package focussing on the main killers of under-five children. It was developed in the 1990’s by the World Health Organization (WHO). IMCI has been associated with a reduction of 13% of under-five mortality in Tanzania and a doubling in the annual rate of reduction of under-five mortality in Egypt (3.3% vs 6.3%) [2, 3]. A 2016 Cochrane review found that IMCI was associated with a 15% reduction in child mortality when activities were implemented in health facilities and communities [4]. IMCI is part of the national child strategy in 90 of the 97 low and middle income countries and is the primary strategy for child health according to WHO [5].

The standard IMCI training package was introduced in Tanzania in 1996 and was given as in-service training over a period of 11 days (standard IMCI). Tanzania had an under-five mortality rate of 58.8 deaths per 1000 live births in 2015 [6]. Scaling up standard IMCI in Tanzania (as in many other developing countries) has faced a number of challenges including low coverage and high cost of training [7–10]. In response to these challenges, the Tanzanian Ministry of Health and Social Welfare (MOHSW) introduced alternative options for IMCI training [11]. The MOHSW developed distance learning IMCI program and started to implement it in 2012, adapting the WHO distance learning curriculum [11–14] in both content and pedagogical approach to the Tanzanian context [2].

This paper describes the evaluation results of distance learning IMCI training program in Tanzania during 2012 to 2015 period. The evaluation includes describing the implementation coverage, clinical skills of HCPs trained in distance learning IMCI versus those trained in standard IMCI, cost of training and perceptions of trainees, trainers and policy makers.

### Distance learning IMCI - a blended learning approach

IMCI, in addition to its current blended training methods, could be improved by adding new methods such as new and improved audio-visuals, SMS messaging, peer training and developing a series of short self-learning modules blended with 3 short face to face orientation or review meetings as we have done in this distance learning IMCI course.

### Course duration and schedule

**Fig. 1:**
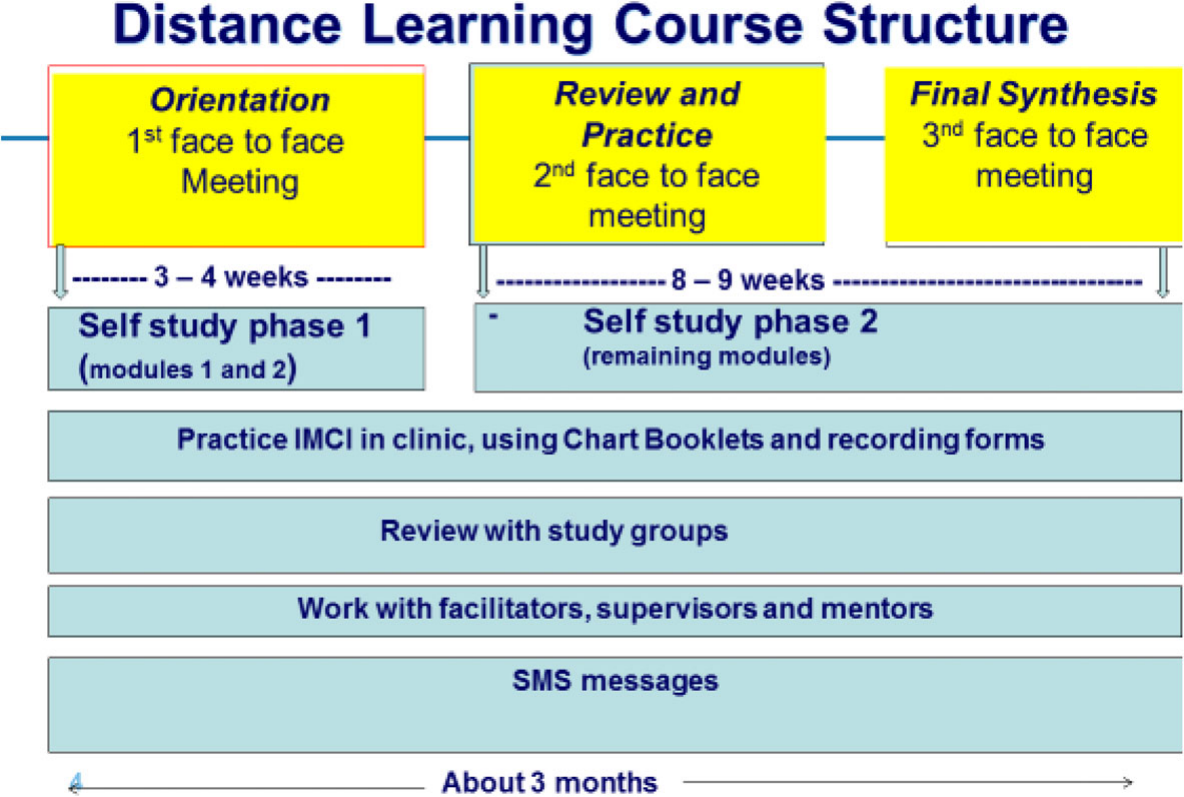
Distance learning IMCI training course - duration and structure

The total duration of the distance learning IMCI course covers a period of 10–12 weeks ([11–14]. Learning is self-directed and self-motivated, with trainees completing modules and exercises as well as clinical practice in their own facilities. The distance learning IMCI course consists of three face-to-face encounters (each about 8 h) between IMCI trainees and IMCI facilitators and two self-study periods (3–4 weeks and 8–9 weeks) (Fig. 1). During the self-study periods trainees maintain contact with facilitators via Short Message Services (SMS) done using mobile phones which each HCP owns.

The first face-to-face meeting is a one-day classroom-based session in which HCPs are oriented to the IMCI approach and provided with guidance about the course including on clinical practice and how to use IMCI DVDs. During the 2nd and 3rd face-to-face meetings, the facilitator reviews their clinical practice and reading of modules. During the self-study periods, facilitators continue to connect with the trainee HCP by SMS and undertake at least one site visit to support learning. At the conclusion of the training period, the trainees take a final examination that they must pass in order to receive a certificate, which is signed by senior officials of the MOHSW.

### Peer learning and SMS messages

The trainees are also assigned to peer groups of 2–5 persons (based on proximity of their health facilities) and given guidance on reading and practicing as a small group in their own set-up. They identify a leader for the group and create an agenda for self-learning and clinical practice. At the conclusion of their meeting, peer members conduct a self-assessment of their IMCI knowledge and skills.

### Follow up visits

Follow-up visits are also conducted 4–6 weeks after training. Follow up visits assess clinical skills, reinforce clinical skills as well as provide supervision on facility support [15]. The follow-up visits also allow the facilitator to assess performance of the HCPs based on the WHO health survey (HFS) indicators [16]. Reports of the follow-up visits, including scoring the results of clinical skills, are copied to the relevant government officials. Facilitators correct any problem related to clinical skills and solve supply issues by ensuring their reports reach the district health office and the MOHSW.

## Methods

In order to assess the performance of distance learning IMCI program which was rolled out beginning 2012, a desk review of reports of all distance learning IMCI courses and reports of follow up visits (Personal communications- F. Bundala, MOHSW)) were made. During follow-up visits, both the clinic and the providers were assessed on a number of criteria including availability of job aides, case management performance based on real and fictional cases and quality of data recording [16].

### Selection of districts

From December 2012 to end of June 2015, a total of 4806 HCPs were trained on distance learning IMCI from 1427 health facilities {HF) in 68 districts in Tanzania. Data on distance learning IMCI participation and results of evaluation during follow up visits were collected in 2013 and 2014. The data presented in this paper is from a selected sample of six districts where the percentage of HCP trained in distance learning IMCI in each district is more than 50% {lringa, Mufindi, Njombe, Makete, Mbarali and Mbeya) as shown in Table 1. These districts had a high coverage of distance learning IMCI courses fulfilling one of the WHO priority indicators for coverage of IMCI [16]. The districts chosen for the standard IMCI are those where IMCI courses were given recently i.e. within 1–2 years of the period the distance learning courses were given so that the HCP had recent and comparable trainings. There were only 5 such districts (Kinondani, Mbagal, Domitilia, Mbeya and Zanzibar) which had the standard IMCI and the number of participants was small. In addition, each course of IMCI in Tanzania enrols similar proportions of HCP from the 3 levels of health care i.e. district hospitals, health centers and dispensaries again providing comparable distribution of HCPs by their work place.

**Table 1:** Coverage indicators in distance learning IMCI in 6 districts

Indicators	Districts					
	Iringa	Mufindi	Njombe	Makete	Mbarali	Mbeya
Total number of Health Facilities (HF) in the District	76	74	25	44	46	65
Number(%) of HF involved in distance learning IMCI training in the District	74 (97)	73(99)	25(100)	35(80)	46(100)	62(95)
Number of HCPs Working with Health Facilities in the district	309	347	192	116	188	262
Number (%) HCPs trained in distance learning IMCI in the district	225 (73)	209(60)	101(53)	137 (100)^a^	153(81)	211(80)
Number of HF visited during this Follow up visit in the district	74	73	25	27	46	62
Number of HCPs (%) distance learning IMCI Trained, found at HF during Follow up visit	138 (61)	109 (52)	56 55)	59(81)	115(75)	105(50)

^a^Note Makete suffered a high turnover of staff as shown by the small number who were working in the facilities compared to those trained in distance learning IMCI earlier- why more than 100% were trained

### Evaluation of case management skills

WHO recommends 18 priority indicators for evaluating IMCI case management skills at health facility level [16]. We selected 4 of these indicators that we believe are representative of all of them for comparing performance of HCPs trained in distance learning IMCI with standard IMCI. The indicators used in this evaluation included skills to recognize sick children who need urgent referral i.e. assessing for Danger Signs, to assess for IMCI Main Symptoms and to treat sick children and counsel caretakers on feeding appropriately. A statistical comparison of proportions of HCPs who performed appropriately was made to test for significance.

### Structured questionnaires to stakeholders

As part of the evaluation the perceptions of trained health care providers, trainers, policy makers and implementing partners were assessed using structured questionnaires and focussed group discussions. Structured questionnaires (Additional file 1) were sent out to 60 facilitators.

The first author drafted the questionnaires and feedback discussions and improvements were made on them by the co-authors. The questionnaires were then tested on a sample of HCP in Dar Es Salaam and improved. They were translated and back-translated to Kiswahili. Most of the questions are closed ended and quantitative proportions could be done even though the numbers were small. Once we had a positive response, we usually get all answers completed or we followed it up.

The questionnaires included experience in IMCI, their perspectives on the contents and approaches of IMCI training, training methods used, their opinions on the effect of IMCI, advantages and disadvantages of distance learning IMCI versus standard IMCI, availability of IMCI materials and tools and challenges in implementing distance learning IMCI versus standard IMCI. Questionnaires were also sent to 20 policy makers, partners, and program managers such as district medical officers (DMOs), regional medical officers (RMIFP), and child health focal persons (Additional file 2). The questions included if they have a strategic plan for implementing IMCI, their opinions on progress of IMCI implementation, barriers to implementation, if they have adapted or adopted distance learning IMCI. The questionnaires were tested on a sample of HCP in Dar Es Salaam, translated and back-translated to Kiswahili before they are used for the evaluation.

### Focus group discussions (FGDs)

Seven focus group discussions (FGDs) were conducted with HCPs to understand their perspective on the different aspects of distance learning IMCI. A list of questions (Additional file 3) were drafted by one of the authors (FB). These were commented upon by other co-authors. Then the list was discussed further with representatives of the seven focus groups and further refined. These revised versions were used in the FGDs and included questions on their experience of IMCI, for how long, what sections they appreciated in the training, how much clinical practice they had, which training approach methods they appreciated most and to grade their confidence in managing sick children. The moderator (FB) opened the discussions with thanking the participants and raised the questions one by one for open discussion for each focus groups. Two note takers were hired to help and assist in data collection.

The target groups for these FGDs were 23 nurses and 23 clinicians who had recently attended (past 6 months) distance IMCI and the IMCI Computer-Based Adaptation and Training Tool (ICATT) [17] training during August and September 2015. Almost all participants received the same learning materials. All participants were mobilized and recruited with assistance from local coordinators. The same moderator and note takers were used for all FGDs. Analysis involved triangulation of the responses for similar questions.

### Statistical analysis

Comparison of performance of HCP between those trained in distance learning IMCI and those trained in standard IMCI was made by comparing proportions. The statistical significance was calculated using Chi square test and a *p* value of < 0.05 was considered significant. The coverage indicators were measured using percentages and did not require testing for statistical significance. A comparison on conducting the training courses for distance learning IMCI versus standard IMCI was based on absolute values of the cost in Tanzanian shillings (also converted to US dollars using exchange rates of the time). Analysis of the interviews were described in percentages for quantitative answers and in the case of qualitative questions or FGDs, commonly expressed perspectives are described.

## Results

### Coverage of distance learning IMCI

**Fig. 2:**
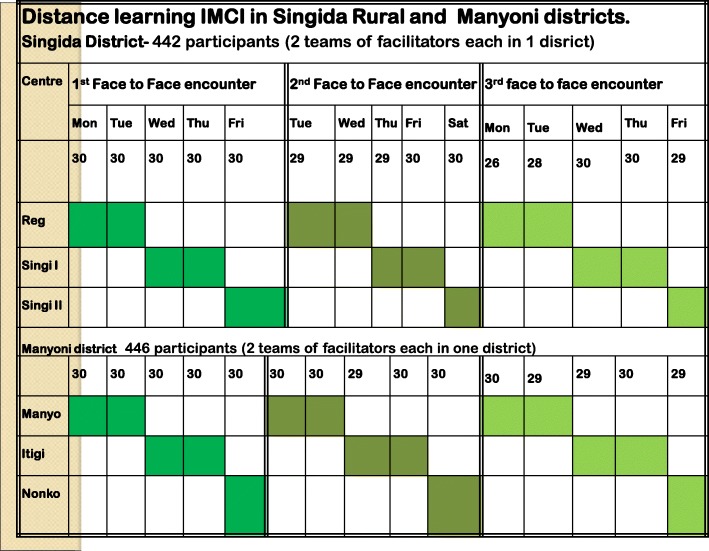
Schedule for clusters of parallel distance learning IMCI training courses in multiple districts per week

Distance learning IMCI allowed clusters of training courses to take place in parallel. This process allowed rapid expansion while reducing facilitator burden because facilitators could travel to a province with courses planned in multiple districts in the same week. This was demonstrated in the two districts of Singida and Manyoni with three training sites, each site accommodating 30 participants every day of the week with same facilitators with an overall training of nearly 900 participants (Fig. 2). HCPs from the same clinic were able to attend the training on different days, ensuring that the clinic remained staffed while all providers had a chance to receive training on different days of the same week.

The coverage of health indicators in the 6 selected districts with more than 50% of HCPs trained in distance learning IMCI is shown in Table 1.

### Case management skills following distance learning IMCI and standard IMCI

Data on performance of HCPs trained in distance learning IMCI from the above 6 districts are compared with performance of HCPs trained in standard IMCI within 1–2 years of the period in the same skills from 5 districts (Kinondani, Mbagal, Domitilia, Mbeya and Zanzibar) as shown Table 2.

**Table 2:** Performance of HCP trained in distance learning IMCI and standard IMCI

Priority indicators for IMCI	Distance learning IMCI districts (*N* = 582)	Standard IMCI districts (*N* = 70)	95% Confidence Interval	Significance level
Proportion (%)HCPs assessed Danger Signs appropriately	89.7	78.6	1.7776 to 22.7754	*P* = 0.006
Proportion (%) HCPs assessed Main Symptoms appropriately	88.6	82.9	−2.7492 to 16.8577	*P* = 0.1661
Proportion (%) HCPs Treated Sick Child Appropriately	76.8	67.1	−1.6683 to 22.4214	*P* = 0.0738
Proportion (%) HCPs Counselled Caretakers on feeding Appropriately	61.5	70.0	−4.2670 to19.6540	*P* = 0.1657

As shown in Table 2, there was no statistically significant difference in the performance of HCPs of those trained in distance learning IMCI compared to those trained in standard IMCI in assessing Main Symptoms, treating sick children appropriately and counselling caretakers on feeding appropriately. There was better performance of those trained in distance learning IMCI in assessing Danger Signs.

### Availability of IMCI job aides

Two of the key job aides that are used by HCPs in IMCI are the chart booklet and the recording forms, which should be present where the HCP is assessing and treating children. The follow-up visit found that all clinics in both distance learning and standard IMCI districts (100%) had the job aides available.

### Cost of conducting training courses: Distance learning IMCI versus standard IMCI

Available cost data suggests that distance learning IMCI is less expensive than standard IMCI, as the distance learning approach requires less venue-related and accommodation-related expenses. The table below shows the costs of standard IMCI training courses for 120 participants and distance learning IMCI training courses for the same number of participants in the same district. The cost of training one participant in standard IMCI and distance learning IMCI is USD 983 and USD 298 respectively - a 70% cost reduction (Table 3). Follow up after training costed around USD 72 per participant, and there was no difference between standard IMCI and distance learning IMCI.

**Table 3:** Comparing cost of training distance learning IMCI versus standard IMCI courses

Components of cost	Standard IMCI		Distance learning IMCI	
	TSH	USD^a^	TSH	USD^b^
Human resources: facilitators	50,400,000	24,585	17,280,000	7680
Human resources: participants	138,240,000	67,434	40,320,000	17,920
Transportation and fuel	5,060,000	2468	2,770,800	1231
Refreshments, conference pack	46,650,000	22,756	15,180,000	6747
Training materials and tools	1,480,000	722	4,920,000	2187
Total	41,830,000	117,966	80,470,800	35,765

^a^One USD exchanged for 2050 Tanzanian shillings (*TSH*)

^b^One USD exchanged for 2250 TSH. The rates for the standard IMCI were lower because the courses were conducted about 1–2 years earlier

### Perspectives of IMCI facilitators, course directors, and clinical instructors

Of the 60 facilitators who were contacted 36 gave responses. Most of the respondents were medical officers or assistant medical officers (25) and nearly all (34) worked in public facilities. Most (64%) had experience facilitating standard IMCI and 92% had experience facilitating distance learning IMCI.

Thirty facilitators (83%) appreciated distance learning IMCI because of the self-learning and peer-learning components. They observed that:

*“Distance learning IMCI is flexible; it frees time for participants (53%); that the additional SMS and DVD video support is very helpful (78%); that learning happens without interrupting services (58%); the clinical practice happens in their own setting (31%)”*

The main advantage of distance learning IMCI cited by the facilitators was the ability to cover a large number of participants. The main disadvantage they cited was that there were not enough guidance for clinical skills and for those who are not self-directed, learning may not be successful; and for slower learners, self-study can be challenging.

### Perspectives of policymakers, partners, and program managers

Completed questionnaires were received from 7 program persons and 3 partners. Of those interviewed, all 10 had a strategic plan for IMCI and were promoting distance learning IMCI. Eight out of 10 had been involved in the process of adapting IMCI to a distance learning format, and all were happy with the content and approach. They observed:

*“The content was “learner-centred”, “comprehensive”, and “easily understood for adult learning”. “The availability of local facilitators to mentor trainees and the in-clinic practice as key to distance learning IMCI’s success””.*

The main advantage of distance learning IMCI cited by the stakeholders was that it increased coverage rapidly, while minimizing cost and time; and that it reduced absenteeism from work stations. The main disadvantage cited was the need for significant supervision and follow-up via SMS.

In terms of barriers to implementation, the most common was funding, followed by inadequate planning and shortage of supplies and medicines. Generally, they felt positively about distance learning IMCI, but felt that more investment was needed to reach the WHO coverage standard of 60% or more of HCPs trained in IMCI in more districts, and felt that more follow-up and oversight was needed.

### Perspectives of HCPs

The clinicians and nurses who participated in the FGDs said that they had all the resources needed (distance learning IMCI chart booklets, distance learning IMCI modules, photograph booklet, recording forms, distance learning IMCI DVD, etc.). However, some clinicians and nurses from rural areas were unable to use the distance learning IMCI DVDs due to lack of electricity or a DVD player. They also said:

“*We enjoyed all modules; however, we feel some modules were more useful than others. For example, we enjoyed the modules on danger signs, cough or difficulty breathing, diarrhoea, fever, anaemia and malnutrition, however, the sections on ear infections and measles were least useful*”

They also said:

“*We had the opportunity to attend to our work and families at home and study simultaneously”.*

*“We had opportunities for day-to-day practice as we attend to patients every day*”.

## Discussion

This is the first study that compares distance learning IMCI with the standard IMCI. While the widespread use of the distance learning approach in the training of HCP appears to be a relatively recent phenomenon, there is growing recognition of the part that it can play in addressing current constraints on human resources for health training in low and middle income countries [18–21]. Experiences have been published in the use of the distance learning approach in various health topics [22, 23].

Our paper suggests that distance learning IMCI is a feasible option especially in settings where funding is a barrier to scale up IMCI. According to the perspectives of stakeholders of IMCI in Tanzania, distance learning IMCI reduces in-person training time needed, allowing facilitators to cluster trainings in a geographic area, maximizing efficiency and rapidly increasing coverage. It causes significantly less disruption to clinical work, as it requires less time out of work than the standard course. It is suitable for private practitioners and those HCPs who need to attend to young children at home and who cannot be away from home for too long. Its structure is flexible to accommodate new modules. Certain modules can be added to the curriculum and implemented in areas of need, as was done with an HIV module for high prevalence districts in Tanzania.

However, there were limitations in our study. The performance of HCPs was not evaluated prospectively; we used data that was collected by facilitators during their follow up visits the results of which could not be controlled. However, the assessors were experienced IMCI facilitators trained to do the follow up visits using the WHO standard procedures and indicators. The comparison group we used i.e. HCP who got trained in the standard IMCI within 1–2 years period were relatively small numbers and this has probably resulted in the wide confidence intervals we showed in the results section.

The response rate to our questions by facilitators, policy makers, program managers and partners was low. However, given the uniformity of the responses provided, we feel, the responses are likely to be reflecting the true picture but we recommend further studies with wider participation.

One of the innovative methods incorporated in distance learning IMCI is peer learning. Unlike the standard IMCI where peer learning is discouraged, in our distance learning IMCI course, it was part of the learning schedules. Peer learning probably contributed to the success of the distance learning IMCI program. Peer learning has been shown to enhance positive changes and has been shown to be a useful adjunct in other areas of clinical skills learning approaches [24, 25].

Assessments of training costs suggest that distance learning IMCI is around one-third to one-half the cost of standard IMCI. The cost savings can be applied to more frequent follow-up visits as well as contribute to the purchase of essential medicines and conducting additional distance learning courses on new nationally identified priority topics such as essential newborn care. The study shows that there is buy-in of the distance learning IMCI program by partners and policy makers in Tanzania who could also contribute to addressing the shortage of supplies and medicines.

## Conclusion

In conclusion, distance learning IMCI is a feasible option to scale up IMCI. It should be accompanied by budgeted support supervision and sustained supply of essential medicines and supplies.

## Additional files

Additional file 1: Questionnaire for IMCI Facilitators. This questionnaire is to be administered to IMCI facilitators, course directors and clinical instructors. (PDF 207 kb)

Additional file 2: Questionnaire for Policy Makers. This questionnaire targets policy makers, partners and program persons including district medical officers and child health focal persons. (PDF 159 kb)

Additional file 3: Guidance on Focused Group Discussions with Health Care Providers. This guidance is for moderators of the FGDs to lead the discussions on IMCI content and approach. (PDF 139 kb)

## Abbreviations

Clinic: First-level outpatient health facility such as a dispensary, rural health post, health centre, or the outpatient department of a hospital
Clinical instructor: A clinician usually practicing in a hospital who teaches IMCI using his/her patients
Counselling caretaker: Skills used in IMCI to teach or advise a mother as part of a discussion which includes: asking questions, listening to the mother’s answers, praising and/or giving relevant advice, helping to solve problems, and checking understanding
Course director: Who takes overall responsibility to run a programme of an IMCI course
Facilitator: Someone supporting learning during a training course
FGD: Focussed group discussions
Follow-up visits: Visit 4–6 weeks after IMCI course usually by IMCI facilitators, course directors, or clinical instructors to reinforce the IMCI skills and to ensure that implementation has started
HCP: Health care provider
ICATT: IMCI computer-based adaptation and training tool
IMCI: Integrated Management of Childhood Illness- Integrated case management process-a process for treating patients that includes consideration of all of their symptoms
Mentor: Someone who supports learning, mostly on the job
MOHSW: Ministry of Health and Social Welfare
Peer group training: A method of training in which a small group learns by reading and doing clinical practice together
SMS: Short Message Service
Support supervision: Visit by members of the district health team to a health facility, usually for administrative support as well as to re-inforce skills
WHO: World Health Organization
