# Supplementary material for: Evaluation of distance learning IMCI training program: the case of Tanzania
Source: BMC Health Serv Res. 2018 Jul 13;18:547. doi: 10.1186/s12913-018-3336-y (PMC6044076; doi:10.1186/s12913-018-3336-y)
Supplement: Supplementary file 1 — Questionnaire for IMCI Facilitators. This questionnaire is to be administered to IMCI facilitators, course directors and clinical instructors. (PDF 207 kb) [file 12913_2018_3336_MOESM1_ESM.pdf]

QUESTIONNAIRE TO BE ADMINISTERED TO

IMCI FACILITATORS/COURSE DIRECTORS/CLINICAL INSTRUCTORS

Instructions:

This questionnaire form is part of a systematic evaluation of IMCI. It is being used to gather in-depth information about the effectiveness of IMCI in improving health care provider's competencies, in particular the distance learning IMCI. Data obtained from this review will be synthesized to improve IMCI training delivery.

•You have been selected to fill in this questionnaire because you are an IMCI facilitator or course director or clinical instructor.

•Your honest assistance and time in completing this questionnaire will thus be 100-fold appreciated.

•The questionnaire has 15 questions over 8 pages.

Although the questionnaire is long, there are several notes and check/tick boxes to assist you in its completion.

•Please answer the questionnaire honestly. Your views will be confidential, and whatever you say/write will help us improve IMCI training.

At the top of each page, please write your unique identifier in the space provided. You can generate this by using the first 3 letters of your country's name, followed by your initials e.g. if you are from GONJA health facility and your name is Anna Sishi Martina then your unique identifier will be GONASM – 1st 3 letters from GONJA and initials A (for Anna), S (for Sishi) and M (for Martina). Make sure that you do this on all the pages and that the identifiers you write on each page are the same.

Note the abbreviations below which you will find in the questions:

sIMCI- standard 11-day IMCI

dIMCI- distance learning IMCI

ICATT- IMCI Computer-based Adaptation, and Training Tool

IMCI FACILITATORS/COURSE DIRECTORS/CLINICAL INSTRUCTORS		
1	**Identification**	
1.1	Name	
1.2	Sex	1. Male 2. Female
1.3	Region	
1.4	District	
1.5	Name of institution	
1.6	Profession	1. Nurse<br>2. Clinical officer<br>3. Assistant medical officer<br>4. Medical officer<br>5. Pediatrician<br>6. Public health officer
1.6	Type of Facility	1. Private<br>2. FBO<br>3. Public
2	**EXPERIENCE IN IMCI**	
2.1	Which of the IMCI training courses have you been trained? (Circle and put year)	1. 11-day IMCI course (sIMCI), Year……<br>2. Computer-based IMCI course (ICATT), Year…..<br>3. Preservice IMCI block or staggered, Year….<br>4. Distance learning IMCI course (dIMCI), Year…
2.2	Which of the IMCI training courses have you been facilitating? (Circle and put year)	1. 11-day IMCI course (sIMCI) , Year……<br>2. Computer-based IMCI course (ICATT), Year…..<br>3. Preservice IMCI block or staggered, Year….<br>4. Distance learning IMCI course (dIMCI), Year…
2.2		
3	**Content of IMCI**	

3.1	Which sections did you appreciate most and which did you not appreciate? Indicate your score in the respective box (Score 5 to 0 for most appreciated to least appreciated).	1. Danger signs ☐<br>2. Cough or difficult breathing ☐<br>3. Diarrhoea ☐<br>4. Fever ☐<br>5. Measles ☐<br>6. Ear infections ☐<br>7. Anaemia ☐<br>8. Malnutrition ☐<br>9. Young infant ☐<br>10. HIV ☐<br>11. Well child ☐
4	**Training methods**	
4.1	As a trainer, what method of training did you enjoy about the IMCI course? (Circle the best three)	1. Classroom<br>2. Clinical practice<br>3. Participatory/group discussion<br>4. Exercise<br>5. Video<br>6. Photographs<br>7. Counselling skills<br>8. Skills of facilitator<br>9. Appropriate pace of teaching
4.2	As a trainer, what method of training did you NOT enjoy about the IMCI course? (Circle the worse three)?	1. Too didactic, too difficult<br>2. Little clinical practice<br>3. Too many exercises<br>4. Too few exercises<br>5. Too much reading<br>6. Too long<br>7. Too short<br>8. Training too hurried<br>9. Training repetitive<br>10. Did not focus on neonates<br>11. Did not focus on HIV<br>12. Video too long or too many<br>13. Video not adequate to demonstrate all the required clinical signs and skills

5	**Effect of IMCI training**	
5.1	What is your opinion on the effect of IMCI on the competency of trained health workers? (Circle the most appropriate)	1. Have not seen much change<br>2. Improvement is very little<br>3. There is improvement on their clinical skills<br>4. There is improvement on their communication skills<br>5. IMCI-trained health workers are motivated
5.2	If you have facilitated both standard IMCI and distance learning IMCI, what is your opinion on its impact on their skills , knowledge and motivation (Circle the most appropriate)	1. dIMCI is equally effective to sIMCI<br>2. dIMCI is better in some aspects to impact on clinical skills<br>3. sIMCI is better in some aspects to impact on clinical skills<br>4. dIMCI is better in motivating trained health workers<br>5. sIMCI is better in motivating trained health workers
6	**IMCI materials**	
6.1	Which IMCI materials/resources do you have during the training courses?	1. IMCI facilitator guide for modules<br>2. Set of IMCI modules<br>3. IMCI chart booklet<br>4. Mother's card<br>5. Photograph booklet<br>6. IMCI video tape/CD/DVD<br>7. IMCI wall chart<br>8. IMCI handbook<br>9. IMCI model chapter for textbooks<br>10. Facilitator guide for outpatient clinical<br>11. Guide for clinical practice in the inpatient ward<br>12. Case recording forms<br>13. Slides for dIMCI<br>14. Others<br>15. Specify_________________________________

7	Challenges during Actual Training	
7.1	What are the challenges you faced during the actual training? If you have done sIMCI	1. Shortage of time,<br>2. Shortage of facilitators<br>3. Shortage of cases for clinical practice<br>4. Shortage of modules<br>5. Trainees are slow to understand<br>6. Shortage of transport<br>7. Shortage of CD's<br>8. Interruption of electricity<br>9. Others<br>10. Specify__________________________
7.2	If you have done dIMCI	1. Shortage of time,<br>2. Shortage of cases for clinical practice<br>3. Shortage of modules<br>4. Trainees are slow to understand<br>5. Shortage of transport<br>6. Shortage of CD's<br>7. Interruption of electricity<br>8. Others<br>1. Specify________
7.3	If you have done ICATT	1. Shortage of time,<br>2. Shortage of cases for clinical practice<br>3. Shortage of modules<br>4. Trainees are slow to understand<br>5. Shortage of transport<br>6. Shortage of CD's<br>7. Interruption of electricity<br>8. Others<br>Specify__________________________
8	Suggestions for improvement to the current training	
8.1	What improvements do you recommend on the existing dIMCI materials? (Circle appropriately)	1. More time is needed<br>2. More facilitators to participant ratio<br>3. All materials should be made available<br>4. Health workers should be provided with mobile phones after training for contacting their facilitators<br>5. Health workers should be provided with money to pay for transport to the group discussion and bills for SMS messages and phones after training for contacting their facilitators.<br>6. Others (specify)……………………………………..

9	**Support you need as a facilitator**	
9.1	What kind of support do you get from your employers?	1. Financial support is provided by organizing agency<br>2. Employer is happy to release me from work<br>3. Employer is NOT always happy to release me from work as sometimes I do not find someone else to cover my own work
10	**IMCI training approaches**	
10.1	Which approach of training do you prefer most?<br><br>Give reasons<br>________________________________<br><br>_______________________________<br><br>_______________________________	1. dIMCI<br>2. sIMCI<br>3. ICATT
10.2	What do you appreciate most about distance learning IMCI?	1. Self-learning and learning with peers (group learning) is good<br>2. Flexible to free time of participant<br>3. Better communication with facilitators via SMS messaging<br>4. HCW can watch DVD during own time and as often as wanted<br>5. Learning is done without interrupting services to patients<br>6. Good clinical practice<br>7. Builds confidence of learning by HCW
10.3	IMCI approach: what do you **NOT** appreciate most about distance learning IMCI?	1. The overall duration of the course is too long<br>2. For some HCWs the actual training has too short a time<br>3. Little guidance on clinical practice

		4. The logbook is too heavy work for some HCWs,<br>5. Some HCWs do not have DVD player to watch the IMCI videos<br>6. Some HCWs do not have mobile phones The examination is too hard for HCWs
11	**Participation in other courses**	
11.1	In what areas have you facilitated trainings in the last 2-3 years	1. PMTCT<br>2. Paediatric HIV<br>3. EID<br>4. Malaria<br>5. Family planning<br>6. Emergency obstetric care<br>7. Essential new born care<br>8. FANC<br>9. ETAT<br>10. Others __________
11.2	Would you have liked them to be given as distance learning?	1. Yes 2. No
11.3	Have you trained your colleagues in the same facility on IMCI?	1. Yes 2. No
12	**Training organized by your own institution**	
12.1	Has your facility organized a training course for its staff?	1. Yes 2. No
12.2	Which areas?(Circle)	1. PMTCT<br>2. Paediatric HIV<br>3. EID<br>4. Malaria<br>5. Family planning<br>6. Emergency obstetric care<br>7. Essential new born care<br>8. KMC<br>9. FANC<br>10. ETAT<br>11. Others _______________
13	**Follow up visits**	
13.1	As a facilitator of IMCI trainings, have you done follow up visits? What is your perspective around follow up visits after IMCI training?	1. I did not do one<br>2. Findings were according to my expectations

		3. Logistically difficult to arrange but worth it because I could see the fruits of the training I gave<br>4. Should be done on reinforcing skills as well as supporting facilities<br>5. Should be accompanied by the DHMT<br>6. Very useful, need to be done more frequently
14	**Barriers to implementation**	
14.1	In your experience which of the following below are the most challenging barriers to implementing IMCI	1. Lack of buy-in from national stakeholders<br>2. Competing priorities<br>3. COST Inadequate funds for training<br>4. Too expensive<br>5. Inadequate fund for printing modules<br>6. HR Lack of facilitators<br>7. Lack of clinical instructors<br>8. Demands too high calibre trainers<br>9. Long duration of course<br>10. Lack of clinical materials.<br>11. Lack of transport
15	**Overall opinion**	
15.1	In your Opinion what do you think are the advantages of distance Learning approach as compared to 11 days training<br><br>______________________________<br>______________________________<br>______________________________	
15.2	In your Opinion what do you think are the disadvantage of distance Learning approach as compared to 11 days training<br><br>______________________________<br>______________________________<br>______________________________	
