# Supplementary material for: Evaluation of distance learning IMCI training program: the case of Tanzania
Source: BMC Health Serv Res. 2018 Jul 13;18:547. doi: 10.1186/s12913-018-3336-y (PMC6044076; doi:10.1186/s12913-018-3336-y)
Supplement: Supplementary file 2 — Questionnaire for Policy Makers. This questionnaire targets policy makers, partners and program persons including district medical officers and child health focal persons. (PDF 159 kb) [file 12913_2018_3336_MOESM2_ESM.pdf]

QUESTIONNAIRE TO BE ADMINISTERED TO

Policy makers, partners and program persons (DMO's, child health focal persons)

QUESTIONNAIRE **– Policy makers, partners and program persons (DMO's, child health focal persons at district, Region and national Levels)**

Instructions:

This questionnaire form is part of a systematic evaluation of IMCI. It is being used to gather in-depth information about the effectiveness of IMCI in improving health care provider's competencies, in particular the distance learning IMCI. Data obtained from this review will be synthesized to improve IMCI training delivery.

The questionnaire has 7 questions over 5 to 6 pages. Please answer all of them.

At the top of each page, please write your unique identifier in the space provided. You can generate this by using the first 3 letters of the name of your institution or organization followed by your initials e.g. if you are from UNICEF and your name is Anna Sishi Martina then your unique identifier will be UNIASM – 1st 3 letters from UNICEF and initials A (for Anna), S (for Sishi) and M (for Martina). Make sure that you do this on all the pages and that the identifiers you write on each page are the same.

Note the following abbreviations:
sIMCI- standard 11-day IMCI
dIMCI- distance learning IMCI
ICATT- IMCI Computer-based Adaptation, and Training Tool

Policy makers, partners and program persons (DMO's, child health focal persons)		
**1**	**Identification**	
1.1	Name	
1.2	Sex	1. Male 2. Female
1.3	Region	
1.4	District	
1.5	Name of institution/organization	
1.6	Area of work (Circle the most appropriate)<br>1. Management/governance<br>2. Public health<br>3. Clinical<br>4. Advocacy /communication<br>5. Other ______________	
1.7	Have you had opportunities to attend in an orientation workshop to IMCI or a full IMCI course<br>1.Yes 2. No	
**2**	**Strategic plan or systematic approach**	
2.1	Is there a strategic plan or a systematic approach to implementing IMCI in your institution/organization/region or district?<br><br>If YES, DESCRIBE the process:<br>______________________________________<br>______________________________________<br>______________________________________<br>______________________________________	1. Yes 2. No
2.2	Is IMCI training in your Institution/organization/region/district systematically linked with other training courses relevant to child survival, such as essential newborn care, kangaroo mother care, infant feeding, EID, PMTCT, emergency obstetric care etc.?<br><br>If YES, DESCRIBE to which training courses?<br>______________________________________<br>______________________________________	1. Yes 2. No

<table>
<tr><td></td><td>____</td><td></td></tr>
<tr><td>2.3</td><td colspan="2">In your opinion, on a scale from -3 to 3 <u>-3= ‘very very slow’ 3= ‘Excellent’</u>
How is IMCI training progressing in the region/district you are working? (ask as per level relevant in question)

<table>
<tr><th>-3</th><th>-2</th><th>-1</th><th>0</th><th>1</th><th>3</th><th>3</th></tr>
<tr><td>Very very slow- couldn’t be slower</td><td>Very slow</td><td>slow</td><td>satisfactory</td><td>Good pace</td><td>Very good pace</td><td>Excellent pace- couldn’t be better</td></tr>
<tr><td></td><td></td><td></td><td></td><td></td><td></td><td></td></tr>
</table>
</td></tr>
<tr><td>4</td><td colspan="2">Barriers</td></tr>
<tr><td>4.1</td><td>What are perceived as the main barriers to rapidly expand IMCI training coverage? Circle the relevant number(es)
<u>(Please provide answers to sIMCI, dIMCI, ICATT , preservice IMCI depending on your experience/ involvement separately).</u>

<u>4.1 If you are promoting sIMCI:</u></td><td><u>4.1 If you are promoting sIMCI</u>
1. Inadequate funding
2. Inadequate planning
3. Inadequate fund for modules, refreshments, accommodation, video, vehicles for transport
4. Shortage of supplies and medicines
5. Shortage of facilitators
6. Competing programs such as PMTCT
7. IMCI course too expensive</td></tr>
<tr><td></td><td><u>4.2 If you are promoting dIMCI</u></td><td><u>4.2 If you are promoting dIMCI</u>
1. Inadequate funding
2. Inadequate planning
3. Inadequate fund for modules, refreshments, accommodation, video, vehicles for transport
4. Shortage of supplies and medicines
5. Shortage of facilitators</td></tr>
</table>

		6. Competing programs such as PMTCT<br>7. IMCI course too expensive
	4.3 If you are promoting ICATT	4.3 If you are promoting ICATT<br>1. Inadequate funding<br>2. Inadequate planning<br>3. Inadequate fund for modules, refreshments, accommodation, video, vehicles for transport<br>4. Shortage of supplies and medicines<br>5. Shortage of facilitators<br>6. Competing programs such as PMTCT<br>7. IMCI course too expensive
5	Facilitators	
	5.1 If you are promoting dIMCI	5.1 If you are promoting dIMCI<br>1. Health workers can learn IMCI without too much interruption of services<br>2. No need for many facilitators like in sIMCI<br>3. Cheaper or less resource internsive<br>4. Many more health workers can be trained in a short time<br>5. Health workers have a greater chance to encounter with more cases for clinical practice<br>6. Others (specify)………..
	5.2 If you are promoting ICATT	5.2 If you are promoting ICATT<br>1. Health workers can learn IMCI in a shorter time

		2. No need for many facilitators like in sIMCI<br>3. Cheaper or less resource internsive<br>4. Many more health workers can be trained in a shorter time and less resources<br>5. Busy Health workers and teachers can learn IMCI at their free time individually<br>6. Others (specify)…………
5	**Adaptation and adoption process of dIMCI**	
5.1	Have you been involved in the process?	1. Yes 2. No
5.2	Do you agree with the process or with the current dIMCI material in terms of content and approach or structure?	1. Yes 2. No
5.3	For Q. 5.2 Give your reasons?<br>____<br>____<br>____	
6	**Performance of dIMCI trained health workers**	
6.1	If you had opportunity to visit these HCW, are you satisfied with their competencies?	1. Yes 2. No 3. Not applicable
6.2	Do you think it is worth the cost/funding you are providing?	1. Yes 2. No 3. Not applicable

6.3	Would you suggest improvements in the current dIMCI training	1. Yes 2. No 3. Not applicable
	If yes, which ones- (circle the most appropriate)	1. Quality of training<br>2. Duration of training<br>3. Number of supervisions<br>4. Number of SMS contacts<br>5. Need additional mentoring<br>6. Need additional distance learning courses to maintain the frequent contact<br>7. Training needs to contribute to career development<br>Other suggestions______________
7	**Overall opinion**	
7.1	In your Opinion what do you think are the <u>advantages</u> of distance Learning approach as compared to 11 days training<br>____<br>____<br>____	
7.2	In your Opinion what do you think are the <u>disadvantages</u> of distance Learning approach as compared to 11 days training<br>____<br>____<br>____	

7.3	Provide any other impression you have on IMCI in general<br><br>____<br><br>____<br><br>____
