# Supplementary material for: Evaluation of distance learning IMCI training program: the case of Tanzania
Source: BMC Health Serv Res. 2018 Jul 13;18:547. doi: 10.1186/s12913-018-3336-y (PMC6044076; doi:10.1186/s12913-018-3336-y)
Supplement: Supplementary file 3 — Guidance on Focused Group Discussions with Health Care Providers. This guidance is for moderators of the FGDs to lead the discussions on IMCI content and approach. (PDF 139 kb) [file 12913_2018_3336_MOESM3_ESM.pdf]

GUIDANCE ON FOCUSSED GROUP DISCUSSIONS WITH

HEALTH CARE PROVIDERS (English translation)

Instructions to moderators

This activity is part of a systematic evaluation of IMCI. It is being used to gather in-depth information about the effectiveness of IMCI in improving health care provider's competencies, in particular the distance learning IMCI. Data obtained from this review will be synthesized and used to improve IMCI training delivery.

You will use this guidance to lead the discussions the discussions. You should allow enough time for participants to provide their opinions. As a moderator you should be listening and managing time at the same time. Your role is to ensure that the discussion topic is introduced and thoroughly addressed, and that the discussion is balanced and inclusive. Avoid expressing your opinion. Be open, alert, probing and encourage everyone to take part in the discussion. You will be accompanied by two note takers who will follow the discussion very closely and take notes.

Topics to be raised for group discussions

1	**Identification**
	Ask participants to provide you their professional level – nurses or clinical officers?
2	**Experience of IMCI**
	Ask participants about their experience in IMCI
3	**Content of IMCI**
	Which sections did you appreciate most and which sections did you appreciate least? Indicate your score in the appropriate box (Score 5 to 1 for most appreciated to least appreciated -5 Excellent, 4 Very good, 3 Good, 2 Satisfactory, 1 Poor)
4	**Clinical practice versus theoretical learning**

	Did you have adequate time in reading the modules?<br>Did you have adequate time to finish exercises?<br>Did you have adequate time to practice on actual patients?
5	**IMCI Teaching /Learning methods**
	What did you enjoy about the IMCI course<br>What did you **NOT** enjoy about the IMCI course- (Circle the worst three)
**6**	**Effect of IMCI training**
	Describe the effect of IMCI training on your performance in clinical practice.
7	**Need to reinforce competency by additional IMCI training**
	Do you think you need to take additional IMCI course to manage sick children because you do not feel confident now?<br>If **Yes**, which one?<br>If **YES,** give the possible reasons for you to need additional IMCI training if you have taken one already?
8	**IMCI training approach**
	What do you appreciate most about distance learning IMCI?<br>IMCI approach: what do you **NOT** appreciate most about distance learning IMCI?<br>What motivates you to complete the dIMCI training (Please give your most honest answer)
9	**Participation in other courses**
	In what other areas have you taken trainings in the last 2-3 years<br>1. PMTCT<br>2. Paediatric HIV<br>3. EID<br>4. Malaria<br>5. Family planning<br>6. Emergency obstetric care<br>7. Essential new born care<br>8. KMC<br>9. Focused Antenatal Care (FANC)<br>10. Emergency Triage Assessment and Treatment (ETAT)<br>11. Other ____________

10	**Peer training**
	Have you trained your colleagues in the same facility on IMCI?
11	**Training organized by your institution**
	Has your facility organized a training course for its staff?<br>Which areas?(Circle)
12	**Follow up visits**
	What is your perspective around follow up visits after IMCI training by your facilitators?
